# Patient involvement in pharmacovigilance: determinants and evolution of reporting from 2011 to 2020 in France

**DOI:** 10.1007/s00228-022-03422-y

**Published:** 2022-12-12

**Authors:** D. Adopo, P. Daynes, M. Benkebil, A. Debs, AP. Jonville-Berra, E. Polard, J. Micallef, P. Maison

**Affiliations:** 1 Department of Pharmacology, Universite Victor Segalen, Bordeaux, France; 2 Agence Nationale de Sécurité du Médicament, Saint-Denis, 93200 France; 3 Pharmacology & Pharmacovigilance Department, Regional Pharmacovigilance Centre of Tours, Tours, France; 4 Pharmacology & Pharmacovigilance Department, Regional Pharmacovigilance Centre of Rennes, Rennes, France; 5 Pharmacology & Pharmacovigilance Department, Regional Pharmacovigilance Centre Marseille Provence Corse, Marseille, France; 6 Faculté de Santé, Université Paris-Est Créteil, Créteil, EA 7379 France; 7 CHI Créteil, Créteil, France

**Keywords:** Patient empowerment, Pharmacovigilance, Adverse drug reaction reporting systems, Drug safety, Social pharmacology, Pharmacoepidemiology

## Abstract

**Introduction:**

Because patients and patient organizations want to strengthen their role in the care pathway and drug evaluation and in order to improve pharmacovigilance activities, European competent authorities implemented regulations to allow direct reporting of adverse drug reactions related to medicinal products by patients in 2012.

**Objectives:**

To describe evolution and analyze determinants of patient reporting activity in France in order to assess patient involvement in pharmacovigilance.

**Method:**

Using the French national pharmacovigilance database, univariate and multivariate analyses were performed to compare the characteristics of adverse drug reaction (ADR) reports from patients and healthcare professionals (HCP) between 2011 and 2020. The relationship between regional patient ADR report activity and regional care provision and socio-professional characteristics was analyzed using the principal component analysis.

**Results:**

A significant and higher increase in ADR reports over time from patients (*r* = 0.89, *p* < 0.001) compared to HCP (*r* = 0.27, *p* = 0.002) has been observed. Patient ADR report activities compared to HCP concerned more women (80% vs. 55%, *p* < 0.001), younger age classes (*p* < 0.001), reporting through web portal (83% vs. 17%, *p* < 0.001), and less serious events (26% vs. 63%, *p* < 0.001). In the principal component analysis, regional patient reporting activity was related to socio-professional categories, age classes, and densities of hospital beds and physicians.

**Conclusion:**

Our results confirm an increasing involvement of patients in ADR report activities. The determinants of patient reporting activities are not only related to drug and medical factors but also to social factors. Digital tools may also play a role in health democracy in pharmacovigilance.

## Introduction

Pharmacovigilance aims to monitor, evaluate, prevent, and manage the risk of adverse drug reactions resulting from the use of the medicinal products [1]. It therefore looks for detection of unexpected adverse drug reaction (ADR) to promote a better use of drugs, thus a reduction of risk for each exposed patient. To achieve this goal, surveillance activity relies on spontaneous ADR reporting, which remains the cornerstone of the early detection of safety signals [2]. Since 1984 in France, the reporting of any adverse reaction that may be due to a drug of which they are aware is mandatory for any physician, dental surgeon, or midwife. This obligation was extended to pharmacists in 1995 [3]. Then, these ADRs are reported to the regional pharmacovigilance centers (CRPV) using the official form [6] and they are evaluated and recorded into the French Pharmacovigilance database (BNPV) since 1985. This anonymized information is transmitted to the French drug agency (ANSM: Agence Nationale de Sécurité du Médicament et des produits de santé) which coordinates the whole process [4]. Since 2011 and 2012, respectively, French and EU pharmacovigilance legislations require all countries across the EU to allow reports directly from patients [1, 5]. In 2017, the French Ministry of Health set up a reporting web portal to enhance vigilance and simplify reporting [6]. Adverse events such as medicinal products can now be reported through a unique tool. Some studies have confirmed the value of patient reporting by describing the medical and pharmacological characteristics of their reports [7, 8], but limited information exists concerning factors leading to patient reporting. Indeed, various determinants may impact ADR reporting, such as media or internet [9–12], for example, the cases of levothyroxine sodium (Levothyrox^®^) and hormonal (levonorgestrel) intrauterine device (HIUD) (Mirena^®^) which proved to be the subject of significant media coverage in 2017 in France [13–15]. In this context, the aim of this study was to assess patient involvement in pharmacovigilance by analyzing the evolution and determinants of patient reporting in France over a 10-year period from 2011 to 2020.

## Methods

### Study design

This is a retrospective comparative study based on data from the French pharmacovigilance database [16] and the following databases: National Institute of Statistics and Economic Studies (INSEE) [17] and Research, Studies, Evaluation, and Statistics Directorate (DREES) [18].

### Data collection

The French pharmacovigilance database contains individual case safety reports (ICSR) reported to the CRPV. These centers are located in the 13 regions of France. These ICSR come from healthcare professionals or patients and patient organizations [16]. Medicinal products are listed as specialty or active substance based on available information. ADRs are coded according to the international terminology of the Medical Dictionary for Regulatory Activities (MedDRA). Our study included spontaneous declarations notified from January 2011 to December 2020. Reports corresponding to follow-up were not considered. For this study, reports from overseas territories were not included, and Corsica was included in the Provence Côte d’Azur region. For each notification, the following variables were extracted: sex, age, region, notification date, notified type, start date, effect type (e.g., medication error and pregnancy), severity, and drugs. Severity means adverse reaction that results in death, is life-threatening, requires hospitalization or prolongation of existing hospitalization, results in persistent or significant disability or incapacity, or is a birth defect.

INSEE is responsible for the production, analysis, and publication of official economics statistics in France [17]. Mean population per region from 2011 to 2020, growth domestic product (GDP) per capita year 2018 per region, and socio-professional categories by region of the year 2017 were collected from INSEE sources [17].

DREES is part of the public statistical service, coordinated by INSEE, and its vocation is to provide reliable information and analyses on populations and health and social policies to public decision-makers, citizens, and economic and social leaders [18]. Physician density and number of inpatient beds per region were collected from DRESS sources.

### Statistical analysis

#### Comparison of patient versus healthcare professional (HCP) reports

A descriptive analysis of the population study on patient (age, sex), report (tool, delay, period, and reporter), and ADR (pregnancy related, medication error) characteristics was conducted and categorized as a healthcare professional or patients.

Descriptive results are expressed either as arithmetic means with standard deviation (SD) or median with interquartile interval (IIQ) or as percentages. The chi2 test was performed to compare the observed percentages. Mean and medians were compared using the Wilcoxon test. Missing data were not considered in the analyses. Multivariate analysis to compare patients versus health professionals was conducted using logistic regression. The independent variables (sex, age classes, severity, reporting modality (web portal versus others: letter, telephone, CRPV website, and CRPV visit), and reporting time) that were significant (p value < 0.2) in the univariate analysis were included in the regression model in which patient versus health professional reports was the dependent variable. The strength of association between the dependent variable and each independent variable was assessed by calculating the adjusted odds ratio (OR) and its respective 95% confidence interval (CI). p value < 0.05 was considered statistically significant.

#### Correlation between patient reporting activity and regional characteristics

The inter-relationship between regional features and patient reporting activity was performed using the principal component analysis (PCA). The PCA [19, 20] is a multivariate method of analysis that explores multidimensional datasets of quantitative variables. It allows to synthesize the information into only a few new variables called components. The Kaiser criterion was chosen to determine the number of axes for the analysis [19]. Only components with a variance or eigenvalue > 1 were retained. Each component was characterized by its loading or correlation with the original variables, which, in this case, were regional reporting activity and regional characteristics (socio-professional categories, gross domestic product, age classes, physician density, and number of inpatient beds). To examine the features to the component, component-loading plots were presented in x-dimensional factor space where x represent the number of components.

#### Evolution of declarations during our study period

The analysis of the change over time (2011–2020) in the number of reports/100,000 was stratified: on the one hand “complete” statements and on the other hand statements without levothyroxine sodium (Levothyrox^®^) and hormonal (levonorgestrel) intrauterine device (HIUD) (Mirena^®^). This second analysis was carried out to take into account possible bias due to the large number of reporting notified in 2017 with levothyroxine and HIUD. Indeed, the analysis of the reporting activity confirmed significant reporting peaks for these products.

#### Top 10 reporting

A top 10 list of the most reported drugs by patients and healthcare professionals was drawn as follows: prior to 2017, from 2017 through web portal reporting and from 2017 via the standard reporting tools (“other”). According to the number of reports, the 10 first drugs were ranked for “patients” and for “healthcare professionals.”

All analyses were performed using SAS^®^ studio software version 9.4.

## Results

### Patients versus healthcare professionals reporting

Table 1 presents and compares patients (n = 67,526) and HCP (n = 371,653) reporting characteristics. Patient ICRS preferably involved cases with females (respectively, 79.5% vs. 54.8%, p < 0.001), younger patient (median of 50 years ± 19 (IIQ: 35–63) vs. 61 years ± 23 (IIQ: 42–75)), and non-serious AEs (74.4% vs 37.1%, p < 0.001). Patients used preferably the web portal to report (82.9% vs. 8.2%, p < 0.001) but reported with longer mean delay (241 ± 757 vs. 150 ± 446 days, p < 0.001).

Multivariate analysis of the reporting characteristics between patient and professional confirms a difference on the female sex (OR = 2.08, CI = 2.02–2.14), the use of the web portal AE reporting (OR = 49.35, CI = 47.89–50.86), and the higher delay for patient reporting (OR = 1.52, CI = 1.49–1.57).

**Table 1:** Characteristics of reporting from patients versus healthcare professionals

	Patients		Healthcare professionals		*p* value
Variables					
	**67,526**		**371,653**		
**Sex**					
Female (*n*, %)	53,671	**79.48**	203,832	**54.84**	< 0.0001
Male (*n*, %)	13,855	**20.52**	167,821	**45.16**	
**Age**					
[0–19] (*n*, %)	5838	**8.78**	31,447	**8.53**	< 0.0001
[20–39] (*n*, %)	15,339	**23.07**	52,736	**14.30**	
[40–59] (*n*, %)	23,787	**35.78**	92,905	**25.19**	
[60–74] (*n*, %)	17,726	**26.66**	97,668	**26.48**	
≥ 75 (*n*, %)	3798	**5.71**	94,119	**25.52**	
**Reporting tools (≥ 2017)**					
Reporting portal (*n*, %)	42,670	**82.91**	8795	**8.21**	< 0.0001
Others (*n*, %)	13,875	**17.09**	155,108	**91.79**	
**Particular circumstances**					
Medication error (*n*, %)	3111	**4.63**	7534	**2.03**	< 0.0001 < 0.0001
Pregnancy (*n*, %)	454	**0.68**	3404	**0.92**	
**Serious adverse event**					
Yes (*n*, %)	17,298	**25.56**	234,753	**62.86**	< 0.0001
**Reporting period (days)**					
Mean	58,846	241 ± 757	343,731	150 ± 445	< 0.0001
Max	–	36,615	–	37,392	
Min	–	0	–	0	

Source: the French pharmacovigilance database from 2011 to 2020. *p* values from Chi² tests and Wilcoxon tests

### Relationship between patient pharmacovigilance activity and region characteristics

**Fig. 1:**
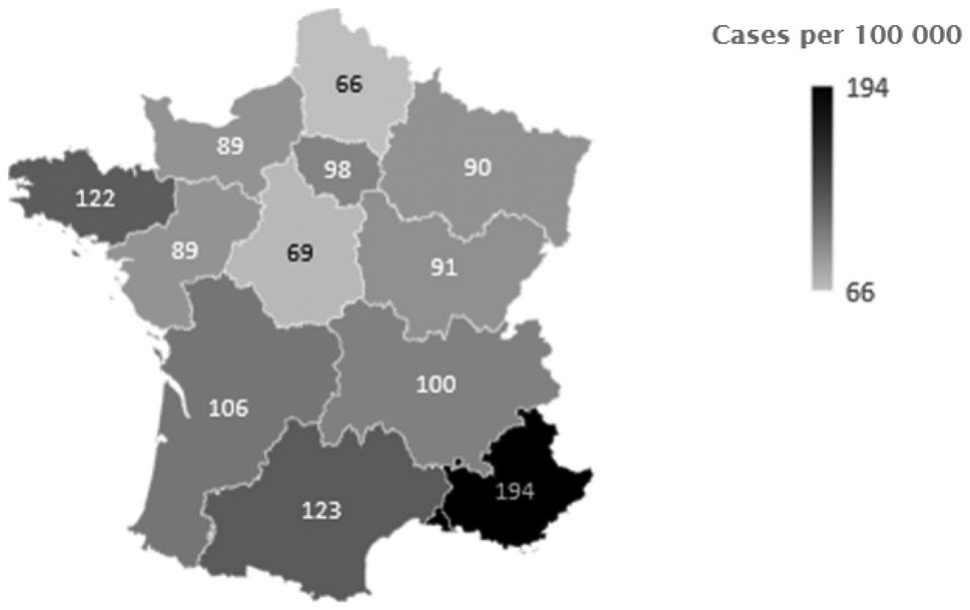
Patient pharmacovigilance activity (number of reported cases) per region and per 100,000 inhabitants

**Fig. 2:**
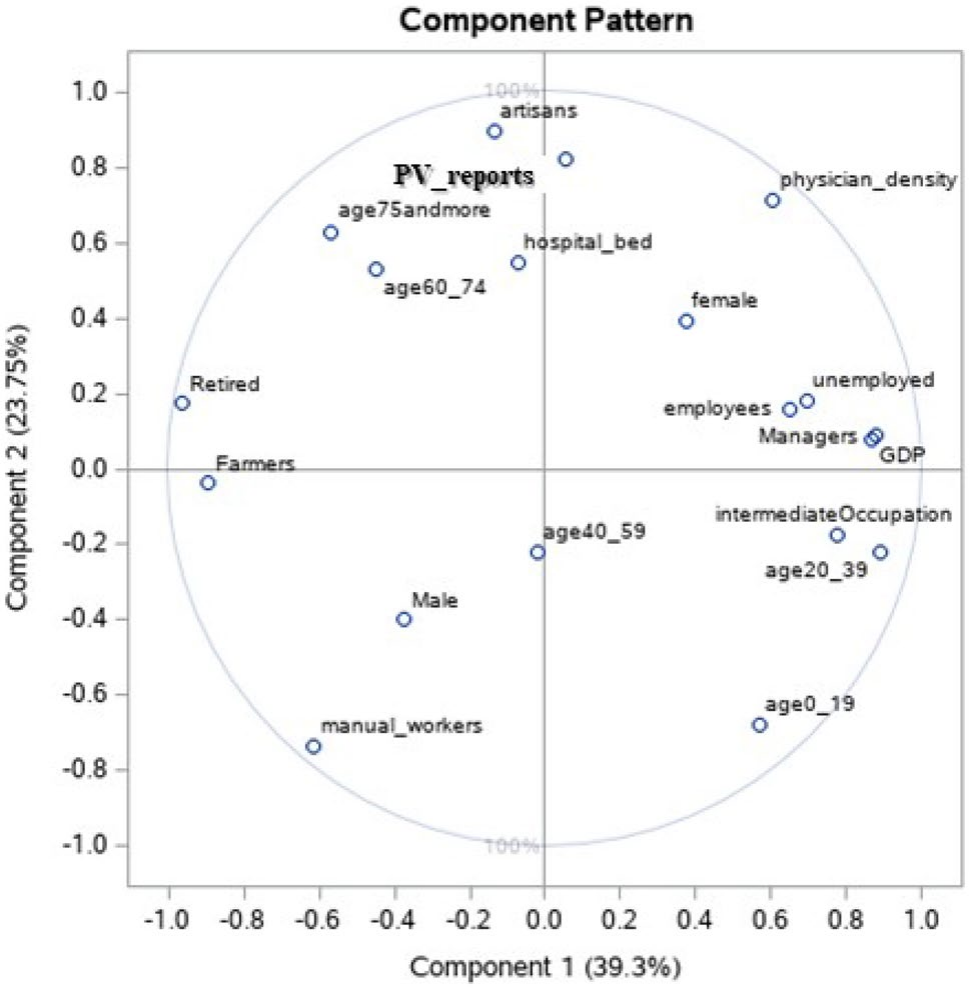
Component-loading plots in two-dimensional space with patient pharmacovigilance activity by region (PV reports) and region characteristics (GDP: gross domestic product; age [0-19]: people aged 0 to 19; age [20-39]: people aged 20 to 39; age [40-59]: people aged 40 to 59; age [60-74]: people aged 60 to 74; age 75 and more: people aged 75 and over)

Apportionment of patient pharmacovigilance activity according to the region is shown in Fig. 1. The results of the PCA are represented with a two-dimensional loading plot (Fig. 2) where each axis shows one component, and the relationship with the regional features is represented by the component loadings. The PCA highlighted two components, one (component 2) of which was particularly correlated with the number of reports per 100,000 residents and positively correlated with physicians’ density and the number of hospital beds, socio-professional classes “artisans,” and age classes (over-75 age group). In contrast, it was inversely correlated with the socio-professional “manual workers” class and the 0–19 age group.

### Evolution of reports

**Fig. 3:**
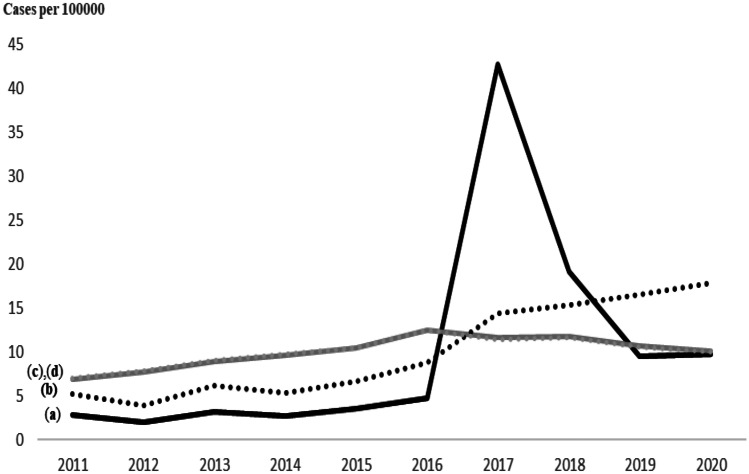
Evolution of adverse drug reporting from 2011 to 2020 with and without levothyroxine sodium and hormonal (levonorgestrel) intrauterine device (HIUD) from patients and healthcare professional (HCP). **a** Patients reports; **b** HCP reports; **c** patients without levothyroxine and HIUD reports (*r* = 0,89; *p* < 0.001); **d** HCP without levothyroxine and HIUD reports (*r* = 0,27; *p* = 0.002)

Figure 3 (curve a) shows an expected peak in patient reporting in 2017 in contrast with HCP reports due to levothyroxine sodium (Levothyrox^®^) and hormonal (levonorgestrel) intrauterine device (HIUD) (Mirena^®^). After removing reports related to levothyroxine sodium and HIUD for all years (curve b), a significant increase in the number of reports per 100,000 residents over time remained (e.g., 6% of reports were observed in 2011 (n = 1585) and 14% (n = 5294) in 2020) with a correlation coefficient of 0.89 (p < 0.001) to patient reports.

### Drugs’ description

Most of the drugs reported in patient reports before 2017 (Table 2) were similar to those reported after 2017 with or without the web portal. However, after 2017, pharmacovigilance activity via the web portal or other processes differed in frequency or in substance. While the drugs involved in HCP reports differed by time period, no differences were observed between reporting tools. But those reported via the web portal were very low.

**Table 2:** Top 10 list of drugs for patient and health professional reports before and after the implementation of the web reporting portal

**Rank**	** < 2017**	*n*	**≥ 2017 via portal**	*n*	**≥ 2017 via “other”**	*n*
			**Patients’ reports**			
**1**	Benfluorex	705	Levothyroxine sodium	28,370	Levothyroxine sodium	6502
**2**	Meningococci type C vaccine	284	HIUD^*^	4173	Meningococci type B vaccine	474
**3**	Tramadol	265	Cyproterone	325	Paracetamol	322
**4**	Paracetamol	250	Tramadol	281	Tramadol	236
**5**	Amoxicillin	226	Levonorgestrel and ethynylestradiol	275	Amoxicillin	226
**6**	Meningococci type B vaccine	225	Desogestrel	274	HIUD^*^	221
**7**	Levothyroxine sodium	197	Acetylsalicylic acid	252	Acetylsalicylic acid	183
**8**	Acetylsalicylic acid	175	Valproic acid	231	Colecalciferol	153
**9**	Ibuprofen	170	Paracetamol	210	Sodium chloride	146
**10**	Esomeprazole	157	Influenza vaccine	206	Cyproterone	144
			**Healthcare professional reports**			
**1**	Acetylsalicylic acid	13,969	Levothyroxine sodium	1229	Paracetamol	11,070
**2**	Paracetamol	12,591	Paracetamol	272	Acetylsalicylic acid	9829
**3**	Fluindione	11,894	Acetylsalicylic acid	211	Furosemide	7510
**4**	Furosemide	10,092	Grippe	169	Levothyroxine sodium	6839
**5**	Esomeprazole	8836	Bisoprolol	166	Bisoprolol	6024
**6**	Bisoprolol	6172	Furosemide	160	Pantoprazole	5786
**7**	Atorvastatin	5739	Pantoprazole	159	Atorvastatin	4868
**8**	Levothyroxine sodium	5724	Atorvastatin	145	Amoxicillin	4394
**9**	Amoxicilline et inhibiteur des betalactamases	5662	Pneumococcus	141	Fluindione	4345
**10**	Enoxaparin	5355	Apixaban	140	Enoxaparin	4290

^*^HIUD: hormonal (levonorgestrel) intrauterine device

## Discussion

Patient reporting is important for pharmacovigilance because it provides additional information not captured by HCP reporting, as previously identified by many studies [21]. In order to assess patient involvement in pharmacovigilance, our analysis aims to identify the determinants and study the evolution of patient reporting activity in France. To our knowledge, this is the first study to suggest that in addition to drug and medical-related factors, demographic and economic factors and digital tools may play a role in patient engagement in pharmacovigilance.

Our results confirm that there is a predominant proportion of females in reports from patient and health professional reports with a statistically higher proportion in patients’ reports. Indeed, previous studies have shown that females are more likely to develop ADRs [22–24]. Similar disparity with HCP has been previously described [25] and could be related to higher healthcare consumption by women [26, 27]. In our study, the proportion of elderly patients was lower in the patients’ reports than in the HCPs’ reports. This may be explained by the fact that older patients have more serious effects leading to hospitalizations [28], and consequently, these ADRs would be reported by professionals. Multivariate analysis tended to confirm this hypothesis since this feature disappears after adjustment for gravity.

Our study also found that patients are more likely to report ADRs related to medication errors or during pregnancy than healthcare professionals. These findings support previous studies’ results highlighting the additional value provided by patient reports [7, 29, 30]. Also, if physicians tend to report major and unexpected ADRs, patients’ reports are more focused on the impact of the ADRs on their daily life [21, 31] and suggesting that HCP may underestimate the burden of minor events for patients [21, 32, 33].

Our results indicating a longer median time to report among patients are consistent with a previous study [32] and suggest different awareness levels about ADR reporting. A review of patient-centered pharmacovigilance published in 2018 highlighted a need for patient education on ADR reporting [34]. Direct patient reporting in pharmacovigilance is still an unknown practice for most patients, basically because they do not know about it or do not feel they are capable of doing it properly [35]. Thus, decision-making and reporting implementation may take longer for the patient.

Regarding the evolution over time of patient reporting, our results show a significant increase in patient reporting from 2017. While a surge in patient reporting of levothyroxine and the hormonal intrauterine device (Mirena®) due to a larger media coverage in France has been previously highlighted [13, 36], our results show that independent to these “reporting surges,” an increasing trend persisted over time. It concerns all products and remains significant after excluding products with media coverage. This increase may suggest a global engagement of patients in their own care and in pharmacovigilance activity independently to media pressure.

The web portal AE reporting setup in 2017 may have facilitated this engagement since in 2017, 82.91% of patient reports are made via the portal. Viard et al. have highlighted that during media crises most patient reports raised from the web portal [14]. This digital tool could be useful to facilitate patient reporting; however, our results did not establish a causal relationship. In addition, barriers to patient reporting activity such as literacy, age, and internet access, persist; therefore, different ADR reporting procedures must remain available.

Regarding the top 10 drugs, those reported by patients tended to be similar over time and with the different tools, although the frequencies were different. The top 10 confirm differences between the characteristics of patient reports and those of healthcare professionals [21, 32, 33]. Both types of statements and their specific features should be considered to improve the management of drugs’ safety.

Our study suggests differences in patient pharmacovigilance activity between regions, and this could be related to regional characteristics such as the density of physicians and the number of hospital beds. The differences in terms of care provision between French regions previously described by Maresca and Helmi [37] could impact pharmacovigilance activity. To a lower extent, this reporting activity was also correlated to socio-professional categories. To our knowledge, this is the first study to suggest a relationship between patient pharmacovigilance activity and care provision or socio-professional determinants.

The main limitation for this secondary analysis is related to the use of retrospective and declarative information and potential recall challenges. Another limitation of our study is related to the official reporting form which does not include professional and socioeconomic information of the reporter. Since individual data were not available, the regional approach may help assess a relationship between these factors and the regional level of patient pharmacovigilance activity. The analysis of the impact of the implementation of the web portal on ADR reporting was mainly limited by the lack of a comparator since it has been set up across the country at the same time. Our data were limited to 2020 because patient reports were primarily focused on anti-COVID-19 vaccines in 2021 [38]. Further analyses are necessary to confirm these hypotheses on the determinants of patient reporting activity.

In conclusion, our study confirms an increasing involvement of patients in pharmacovigilance reporting activity over the 2011–2020 period. The determinants of patient reporting activities are not only related to drug and medical factors but also to care provision and socio-professional factors. Digital tools may also influence patient reporting and facilitate health democracy in pharmacovigilance.

## Data Availability

The data that support the findings of this study and publicly available are online: https://www.insee.fr and https://drees.solidarites-sante.gouv.fr.
